# Investigating the Association of Assisted Reproduction Techniques and Adverse Perinatal Outcomes

**DOI:** 10.3390/jcm13020328

**Published:** 2024-01-06

**Authors:** Anastasios Potiris, Paraskevas Perros, Eirini Drakaki, Despoina Mavrogianni, Nikolaos Machairiotis, Antonios Sfakianakis, Theodoros Karampitsakos, Dionysios Vrachnis, Nikolaos Antonakopoulos, Periklis Panagopoulos, Peter Drakakis, Sofoklis Stavros

**Affiliations:** 1 Third Department of Obstetrics and Gynecology, University General Hospital “ATTIKON”, Medical School, National and Kapodistrian University of Athens, 124 62 Athens, Greece; nmachai@med.uoa.gr (N.M.); theokarampitsakos@hotmail.com (T.K.); pdrakakis@med.uoa.gr (P.D.); sfstavrou@med.uoa.gr (S.S.); 2 First Department of Obstetrics and Gynecology, Alexandra Hospital, Medical School, National and Kapodistrian University of Athens, 115 28 Athens, Greece; paris_per@yahoo.gr (P.P.); eirinidrak@med.uoa.gr (E.D.); dmavrogianni@med.uoa.gr (D.M.); 3 London Women’s Clinic, Marylebone, London WIG 6AP, UK; 4 Medical School, National and Kapodistrian University of Athens, 115 27 Athens, Greece; dvrachnis@med.uoa.gr; 5 Department of Obstetrics and Gynecology, University of Patras, 265 04 Patras, Greece; antonakopoulos2002@yahoo.gr

**Keywords:** assisted reproduction techniques, perinatal outcome, pre-term birth, congenital anomalies, genital defects, heart defects

## Abstract

Background: Infertility affects about 80 million individuals worldwide and 10–15% of couples at reproductive age will seek medical assistance. There is increasing evidence that pregnancies after Assisted Reproduction Techniques (ART) are associated with pre-term birth, low birthweight, congenital defects, and increased mortality rates. The aim of this review is to assess all the published literature and provide an updated review on the effect of assisted conception and perinatal and neonatal outcomes. Methods: Comprehensive research on Pubmed/Medline, Scopus, and Google scholar electronic databases was conducted from July 2023 up to September 2023, using the terms assisted reproductive techniques, ART, in vitro fertilization, IVF, intracytoplasmic sperm injection, ICSI, preterm birth, PTB, low birth weight, LBW, chromosomal defects, congenital defects, and hypospadias. In total, 87 full text articles were retrieved and after a careful evaluation, 31 studies were selected for data extraction. Results: Our review demonstrated a higher risk of congenital and chromosomal defects, and a higher incidence of male genital tract defects and heart defects in ART pregnancies. Regarding pre-term birth, our results were contradictory. Conclusions: Although assisted reproduction techniques are associated with increased risks, they are safe regarding perinatal outcomes and couples should not be discouraged from utilizing them. Our results aim to alert clinicians to these specific outcomes and offer more personalized care and counseling to infertile couples and their children.

## 1. Introduction

A couple is considered infertile when they are unable to conceive after 1 year of unprotected intercourse. Infertility affects about 80 million individuals worldwide and in the United States it is estimated that 12% of the couples at reproductive age will seek medical assistance [1]. An estimate of at least 12 million births have resulted from assisted reproduction techniques, as announced by ICMART (International Committee Monitoring Assisted Reproduction Technologies) in the European Society of Human Reproduction and Embryology (ESHRE) meeting in 2023. Incorporating techniques such as intracytoplasmic sperm injection (ICSI) and testicular sperm extraction (TESE), the rate of Assisted Reproduction Technique (ART) births is expected to surpass 4% of total births [2,3,4].

According to ICMART, each years’ ART cycles rise by approximately 6.7%. Hence, the crude number of pregnancies resulting from ART increases worldwide [5]. Consider, also, that modern ways of life and socioeconomic factors further delay childbearing, resulting in a poorer quality of oocytes and semen. This further enhances the need to assess the association of ART with perinatal and neonatal outcomes [6,7].

ART-conceived pregnancies are associated with an increased risk of twin or multiple pregnancies, which have a six-fold increased risk for prematurity and severe perinatal outcomes and increased mortality [8,9]. The higher risk of multiple pregnancies has led to the initiation and adoption of single embryo transfers (sET) [10]. Nevertheless, there is increasing evidence that singleton pregnancies after ART also are associated with pre-term birth, low birthweight, congenital defects, and increased mortality rates [1,11]. Permanent controversy exists for the origin of possible elevated rates of adverse obstetric and perinatal outcomes. It can either be attributed to the underlying infertility itself or to the assisted reproductive techniques.

The aim of this review is to assess all the published literature and provide an updated review on the effect of assisted conception and perinatal and neonatal outcomes. Specifically, we investigated the impact of ART pregnancies in preterm birth, low birth weight, congenital disorders, urological disorders, and metabolic disorders.

## 2. Materials and Methods

We conducted comprehensive research on Pubmed/Medline, Scopus, and Google Scholar databases from July 2023 up to September 2023, using the terms assisted reproductive techniques, ART, in vitro fertilization, IVF, intracytoplasmic sperm injection, ICSI, preterm birth, PTB, low birth weight, LBW, chromosomal defects, congenital defects, and hypospadias. These keywords were either used separately or in combination with the help of the Boolean administration (OR, AND). All articles with an English title and abstract were initially accepted, irrespective of the time of publication and full-text availability. Through the initial research stage, 362 publications were retrieved. Titles and abstracts of the retrieved articles were assessed by two independent reviewers, P.P. and A.P. If a study was selected by only one reviewer, the decision was taken by a third reviewer, S.S.

**Figure 1:**
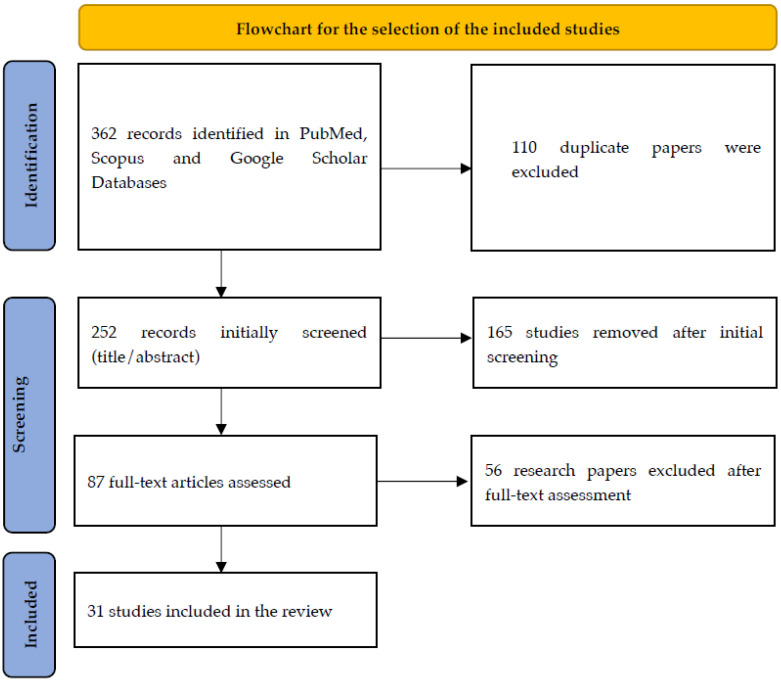
Flowchart of the selection process.

In total, 87 full-text articles were retrieved and, after evaluation, thirty-one studies were selected for data extraction. Our inclusion criteria contained only original articles, case reports, cohort studies, and case series. Articles in animal models, systematic reviews, and metanalyses, or in a language other than English, were excluded. The stages of the article selection process are presented in the diagram in Figure 1.

## 3. Results

There are a variety of published studies comparing newborns conceived with or without assisted reproduction techniques. In this section, we present our study’s results regarding the outcome, namely, pre-term birth and birthweight, chromosomal and genetic defects, urological disorders and hypospadias and, lastly, heart and metabolic defects.

### 3.1. Pre-Term Birth and Birthweight

Numerous studies evaluate the effect of ART in perinatal outcomes. This subsection summarizes our results regarding gestational age at delivery, risk of pre-term birth, and birthweight. Tommasso et al. studied all early preterm deliveries in Florence (2010–2017), including 71 ART pregnancies and 640 conceived spontaneously [12]. The authors found no difference regarding low birthweight comparing the two groups. Similarly, umbilical artery pH, Apgar score at 1 and 5 min, intrauterine growth restriction (IUGR), cholestasis of pregnancy except for caesarean delivery, placenta previa, and pregnancy-induced hypertension (PIH) disorders had no significant difference in ART and spontaneously conceived pregnancies. Similar conclusions were affirmed by Simpson et al., who focused mainly on ICSI, a treatment proper for mild to severe male infertility or cases of infertility unable to be treated by classic IVF. Their study included 6077 ICSI and showed no statistically significant difference regarding pre-term deliveries [13]. Furthermore, Marconi et al. compared in vitro fertilization (IVF)/intracytoplasmic sperm injection (ICSI) blastocyst-stage (*n* = 11.152) and cleavage-stage embryo transfer cycles (*n* = 55.995), ending with comparable outcomes regarding the risk of pre-term birth (PTB) and high birth weight (HBW) [14]. Sunkara et al. published their study about the possible association between ovarian stimulation following IVF treatment and risk of preterm birth (PTB) and low birth weight (LBW) [15]. The authors included data from the Human Fertilization and Embryology Authority (HFEA), for all ART cycles in the UK from 1991 to 2011. Comparing 584.835 stimulated IVF cycles and 6168 unstimulated, there was no significant difference in the risk of the adverse perinatal outcomes after adjusting for potential confounders. A year later, Sunkara et al. investigated whether preimplantation genetic diagnosis (PGD) is associated with the risk of adverse perinatal outcomes, especially PTB and LBW [16]. The study consisted of 87.571 singleton live births following autologous stimulated IVF ± ICSI and 439 singleton live births following PGD. The data collected were also from the HFEA registry. As a result, there was no increase in the risk of adverse perinatal outcomes of PTB and LBW in the PGD and ART group. Premru-Srsen T. et al., in a cohort study involving 761 infertile women who conceived the following year after reproductive surgery (333 after ART cycle and 428 spontaneously conceived) compared to 758 age-matched controls, showed similar results [17]. Additionally, no significant difference regarding PTB between ART and the control group was reported by Scherrer et al. [18].

On the other hand, Szymusik et al., in their case–control study in a Caucasian population, showed that the ART group had a two-fold increased risk for pre-term birth as compared with age-matched controls and a 2.2-fold increased risk for low birthweight [19]. Kaveh et al., in their research, report a significant increase in pre-term labor and the premature rupture of membranes (PROM) in the ART group [20]. The ART group also demonstrated increased rates of intensive care unit admission and pregnancy-induced hypertension. Al Fifi et al. also reported an increased risk of pre-term births in the ART group in their case–control study [21]. Increased risks of PTB and LBW were also reported by Kamath et al. [22]. Furthermore, their study pointed to the possible risk of pregnancy complications after using donor oocytes compared to autologous IVF. Lastly, Sunkara et al. reported increased risk for adverse perinatal outcomes in pregnancies after excessive ovarian response when they compared to normal or poor responses and general population incidence [23].

Generally, pregnancies after ART are considered to be high-risk pregnancies. However, in our study there are controversial results. There were five articles [19,20,21,22,23] pointing to the increased risk of preterm births and another seven that showed no statistically significant outcome regarding preterm births [12,13,14,15,16,17,18] (Table 1). More analytically, most of the studies that compared ART groups with the general population found an increased risk of PTB and LBW [19,20,21]. On the other hand, studies that compared different assisted reproduction techniques presented comparable outcomes [13,14,15,16]. The inconsistent findings appear to be primarily linked to the ART procedures, the sample under study, and the cause of infertility.

### 3.2. Congenital and Chromosomal Defects

Yuan et al. compared 1496 fetuses after IVF/ICSI versus 1396 fetuses from natural conception and displayed a slight but not statistically significant increase in the de novo chromosomal anomalies in the ART group [24]. Marconi et al. compared blastocyst-stage and cleavage-stage embryo transfer cycles and showed a 16% higher incidence of aneuploidies in the blastocyst-stage group [14]. A similar incidence of congenital disorders was also reported by Al Fifi et al. and Olson et al. [21,25]. Both studies reported comparable rates of major birth defects.

A population study by Luke et al. showed higher rates of congenital defects in ART-conceived pregnancies compared to the general population, especially in frozen/thawed cycles [26]. Moreover, Fauque et al., analyzing 3.5 million live births, found a higher prevalence of genetic disorders in pregnancies after fresh embryo transfers or frozen ART cycles [27]. Statistically significantly elevated rates of congenital disorders were also reported by Belva et al. [28].

Another study by Jozwiak et al., from 1136 ICSI pregnancies, found no difference regarding adverse genetic outcomes [29]. On the other hand, Samli et al. and Simpson et al. both found a higher incidence of congenital defects in the ICSI group [13,30]. Table 2 summarizes the included studies regarding assisted reproduction techniques and congenital defects.

### 3.3. Hypospadias—Male Genital Anomalies

Male genital anomalies are considered to be related to ART, especially hypospadias and cryptorchidism. Hypospadias is a condition characterized by the incomplete or ineffective formation of the urethral folds and affects about 0.3% of total live births. Progesterone intake during pregnancy has been associated with an increased risk of developing hypospadias [31]. In this section, we present studies referring to male genital anomalies to establish whether an association with ART exists.

Silver et al. reported a five-fold increased risk for hypospadias in pregnancies after ART [32]. Their retrospective study compared children conceived after ART to those spontaneously conceived. The authors denote that increased exposure to progesterone in the ART group might be the cause of the higher incidence. Similar results are also reported by Funke et al., with a three-fold increased risk for hypospadias but not cryptorchidism [33]. Additionally, Bang et al. reported increased risks in the ART group for both hypospadias and cryptorchidism [34]. Moreover, the authors associated the presence of urological defects with increased risks for pre-term birth and low birthweight. A statistically significant association regarding hypospadias and ART was also shown by Simpson et al. [13]. On the other hand, Aliani et al. reported no significant difference for any genital defect, among ART and ICSI especially [35]. All the aforementioned studies are presented in Table 3.

### 3.4. Heart and Metabolic Defects

Scherrer et al., in their cohort study, evaluated the systemic and pulmonary vascular function in ART pregnancies [18]. The authors report a 25% smaller brachial artery and a 30% increased pulmonary artery pressure in the ART group. Similar adverse outcomes are reported by Liu et al. Their observational study showed affected systolic contraction and diastolic dysfunction in children after ART at the age of five [36]. On the other hand, Arx et al. reported no significant difference regarding cardiac function and pulmonary artery pressure as evaluated via echocardiography [37]. Lastly, cardiovascular defects were also increased in the ART group, in a retrospective cohort conducted by Olson et al. The study showed an increased systolic and diastolic BP and generally affected heart development and function among adolescents [25].

Ceelen et al., evaluating the metabolic profile of ART group children, reported a higher percentage of body fat and comparable rates of bone mineral composition in the aforementioned group [38]. A year later, a cohort study regarding the cardiometabolic status of ART offspring from the same center revealed an increased systolic and diastolic blood pressure (BP) and a higher fasting glucose level in the ART group [39]. The metabolic profile of ART children was also studied by Sakka et al. Comparing 106 ART and 68 spontaneously conceived children, the authors report statistically significantly higher systolic and diastolic BP and triglyceride levels in the ART group [40]. Table 4 summarizes the included studies regarding heart and metabolic defects.

## 4. Discussion

This review summarizes the possible perinatal and neonatal outcomes in pregnancies after assisted reproduction techniques. More specifically, we analyzed the effects of ART regarding pre-term births, low birthweight, congenital defects, male genital anomalies, and heart and metabolic defects.

Regarding pre-term births and low birthweight, the results of the published literature are contradictory. We presented five studies [19,20,21,22,23] reporting an increased risk for preterm birth and seven that showed similar outcomes compared to the general population incidence [12,13,14,15,16,17,18]. It is important to highlight the equal outcomes among ART pregnancies after blastocyst and cleavage-stage embryo transfers [14]. Similarly, PGD also does not affect pregnancy outcomes regarding pre-term birth and low birthweight [16]. An interesting report from Sunkara et al. showed that excessive ovarian response (>20 oocytes) was associated with increased risk for pre-term birth [23]. It is worth mentioning that our review included only original research articles. There are published reviews and systematic reviews in the literature pointing towards a higher incidence of preterm birth and low birthweight in ART pregnancies. Pandey et al. included in their review 20 matched and 10 unmatched cohort studies and showed that IVF/ICSI pregnancies had a higher risk for congenital anomalies (RR, 1.67, 1.33–2.09), the preterm rupture of membranes (RR, 1.16, 1.07–1.26), low birthweight (RR, 1.65, 1.56–1.75), and preterm delivery (RR, 1.54, 1.47–1.62) [5]. Similarly, Qin et al., in their two reviews, included more than 50 cohort studies with more than 160000 ART pregnancies and over 2 million controls. In both reviews, the data analysis revealed that ART pregnancies are associated with an increased risk of developing congenital malformations, low birthweight, and preterm birth [41,42]. Moreover, Cavoretto et al. studied 8044 IVF/ICSI pregnancies in comparison with 53633 spontaneously conceived singleton pregnancies. The authors showed that IVF/ICSI pregnancies have a higher incidence for spontaneous preterm birth before 37 weeks (OR, 1.63; 95% CI, 1.30–2.05) and before 34 weeks of pregnancy (OR, 1.78; 95% CI, 1.03–3.08) [43]. However, the quality of evidence was low and very low for preterm births before 37 and 34 weeks, respectively.

As far as chromosomal and genetic defects are concerned, the results of the literature were more conclusive. Most of the studies report an increased risk of ART offspring for genetic abnormalities [13,25,26,27,28,29,30]. There were also studies pointing to either a slight but not significant increase in genetic defects or no difference at all [14,21,24]. It is interesting to note that ICSI pregnancies, particularly, are associated with the highest risk for chromosomal anomalies and de novo genetic defects. Poor semen qualitative parameters or hidden parental disorders such as mosaicisms might offer a possible explanation for the increased risks of ICSI pregnancies. Morel et al. emphasize the need for parental karyotyping, prenatal counseling, and further screening tests in those infertile couples with severe male factor [44].

Regarding male genital anomalies, and specifically hypospadias and cryptorchidism, there is a conclusive increased incidence in newborns after ART [13,32,33,34,35]. This should alert clinicians to thoroughly examine embryos and newborns for genital track abnormalities in ART pregnancies. The latter is crucial in cases of cryptorchidism since it is associated with increased risk for testicular cancer and a predisposition for infertility [45]. Furthermore, the excessive use of progesterone during fresh and frozen/thawed cycles might also be related to the increased incidence of hypospadias [31,32].

Regarding heart and metabolic defects, our review showed that ART offspring are at increased risk for elevated blood pressure in childhood and cardiac remodeling [18,36,38,39,40]. Only one study showed a larger right ventricular end-diastolic area without increased blood pressure [37]. Similarly, the metabolic profile of ART children was also affected compared to spontaneously conceived children. One study of our review showed increased triglyceride levels in the ART group [40]. The altered metabolic profile can lead to increased BMI (overweight and obesity) and further predispose these children to cardiovascular disorders in adult life [46]. However, the underlying pathways that lead to both genital and heart and metabolic disorders are not yet clarified, and further research is necessary.

It should be mentioned that our review included studies with a high diversity of study samples, different geographic regions, races, socioeconomic status, and time periods. Studies from low-income countries or older periods might impact the access to fertility treatments availability. Moreover, older studies utilized different ART protocols and different medications. Furthermore, newer studies also included women with advanced maternal age, which might also have an impact on perinatal outcomes. Further prospective studies are needed to investigate the potential pathways and underlying mechanisms that are associated with the increased male genital tract anomalies and the heart and metabolic defects of ART offspring.

## 5. Conclusions

Our review demonstrated a higher risk of congenital and chromosomal defects, and a higher incidence of male genital tract defects and heart defects. Regarding pre-term birth, we presented five studies reporting increased incidence in ART pregnancies and seven reporting similar outcomes. Hence, more studies are needed to clarify the impact of assisted reproduction techniques on preterm birth. All these factors have been studied separately, and to our knowledge our review is the most updated and comprehensive. Our limitations include the diversity of each study sample, with different geographic regions, races, socioeconomic status, and fertility treatment availabilities. It should be stressed that although assisted reproduction techniques are associated with increased risks, they are safe regarding perinatal outcomes; couples should not be discouraged from utilizing them. Our results aim to increase the vigilance of obstetricians and pediatricians to these specific outcomes, offering more holistic and personalized care and counseling to infertile couples and their children.

## Figures and Tables

**Table 1:** Studies included in the review regarding pre-term birth and low birthweight.

	Year	Design	Sample		Outcome	Mean ± SD	CI
			ART	Control Group			
Tommaso et al. [12]	2010–2017	Retrospective case–control study	71	640	No difference		
Simpson et al. [13]	1997	Prospective cohort study	6077	General population	No difference		
Marconi et al. [14]	1991–2012	Retrospective cohort study	11.152 Blastocysts vs. 55.995 cleavages	General population	No difference at the risk of preterm birth	PTB: 1 (0.79–1.25) LBW: 0.92 (0.73–1.16)	99.5%
Sunkara et al. [15]	1991–2011	Retrospective cohort study	584,835 Stimulated IVF cycles	6168 unstimulated	No significant difference	PTB: 1.27 (0.8–2) LBW: 1.48 (0.9–2.42)	95%
Sunkara et al. [16]	1996–2011	Retrospective cohort study	439	87.571	No difference	PTB: 0.68 (0.46–0.99) LBW: 0.56 (0.37–0.85)	95%
Premru-Srsen T. et al. [17]	2012–2015	Retrospective case–control study	333	1186	Increased risk but not statistically significant	PTB: 1.07 (0.63–1.81)	95%
Scherrer et al. [18]	2007–2010	Prospective cohort study	65	57	No increase in PTB and LBW risk		
Szymusik et al. [19]	2004–2014	Retrospective case–control study	336	308	Increased risk for PTB and LBW	PTB (OR = 2.06; 1.16–3.68) LBW: (OR = 2.27; 1.19–4.36)	95%
Kaveh et al. [20]	2004–2009	Retrospective case–control study	84	106	Significant higher risk for preterm labor	PROM: 0.2 (0.07–0.8) LBW: 1.4 (0.6–3)	95%
Al-Fifi et al. [21]	2003–2007	Retrospective case–control study	327	354	Shorter birth date at ICSI group.		
Kamath et al. [22]	1991–2011	Retrospective case–control study	5929	127.856	Higher risk for PTB and LBW	PTB: 1.54 (1.34–1.80) LBW: 1.43 (1.24–1.66)	99.5%
Sunkara et al. [23]	1991–2008	Observational study	65.868 live births after ART	General population	Significantly higher risk of PTB and LBW in the study	PTB: 1.15 (1.03–1.28) LBW: 1.17 (1.05–1.30)	95%

**Table 2:** Studies included in the review regarding congenital defects.

	Year	Design	Sample		Outcome	Mean ± SD	CI
			ART	Control Group			
Marconi et al. [14]	1991–2012	Retrospective cohort study	11.152 Blastocyst-stage vs. 55.995 cleavage-stage	General population	16% higher risk in blastocyst stage group	0.9–1.49	99.5%
Al-Fifi et al. [21]	2003–2007	Retrospective case–control Study	327	354	No difference for major defects		
Yuan et al. [24]	2004–2020	Retrospective cohort study	1496	1396	Slight but not statistically significant increase	1.03 (0.71–1.5)	95%
Olson et al. [25]	1989–2002	Retrospective cohort study	1805	8422	Slightly higher rate of major birth defects	1.30 (1–1.67)	95%
Luke et al. [26]	2004–2017	Population-based cohort study	165.125 ART, 12.451 OI/IUI	1.353.440	Higher risk	1.22–1.85	95%
Fauque et al. [27]	2013–2017	Retrospective cohort study	20.218 IUI, 45.303 fresh-ET 18.885 FET	3.417.089 from NC	Increased risk in fresh-ET and FET group.	Fresh-ΕΤ: 1.15 (1.1–1.2) FET: 1.13 (1.05–1.21)	95%
Belva et al. [28]	2004–2012	Prospective clinical follow-up study	1114	General population	Abnormal fetal karyotype was found 41/1114	3.7% (2.7–4.9%)	95%
Jozwiak et al. [29]	1997–2002	Retrospective case–control study	1136	General population	No difference between ICSI group due to male factor and other subfertility issues		
Samli et al. [30]	1996–2000	Prospective cohort Study	142	General population	Increased rate of genetic defects in ICSI pregnancies		

**Table 3:** Studies included in the review regarding male genital anomalies.

	Year	Design	Sample		Outcome	Mean ± SD	CI
			ART	Control Group			
Simpson et al. [13]	1997	Prospective cohort Study	6077	General population	Increased risk for hypospadias	2.9 (1.4–5.4)	95%
Silver et al. [32]	1988–1994	Retrospective case–control study	14	14	5x higher risk in IVF group versus the control group	1.46% IVF 0.27% control	
Funke et al. [33]	1999–2008	Retrospective case–control study	890	14316	Increased risk for hypospadias; not cryptorchidism	3.19 (1.266–8.042)	95%
Bang et al. [34]	2008–2011	Prospective cohort study	7752	General population	Increased risk in the ART group	99 (1.3%) cryptorchidism 8 (0.1%) hypospadias 4(0.05%) both	
Aliani et al. [35]	2013–2015	Prospective cross-sectional study	5608	General population	No relationship with infertility factor	0.34% cryptorchidism 0.038% hypospadias	

**Table 4:** Studies included in the review regarding heart and metabolic defects.

	Year	Design	Sample		Outcome	Mean ± SD	CI
			ART	Control Group			
Scherrer et al. [18]	2007–2010	Prospective cohort study	65	57	ART group: 25% smaller brachial artery, 30% higher pulmonary artery pressure.		
Liu et al. [36]	2015	Prospective observational study	100	100	Higher rates in systolic and diastolic heart disorders		
Arx et al. [37]	2015	Prospective clinical trial	54	54	RV end-diastolic area significantly larger in study group; no difference in pulmonary artery pressure		
Ceelen et al. [38]	1986–1995	Retrospective cohort study	233	233	Higher body fat percentage in study group		
Ceelen et al. [39]	1986–1995	Retrospective cohort study	1313	131	Elevated rates of BP and blood glucose in ART group	Systolic BP: 2.1 (1.4–3.3) Diastolic: 1.9 (1.2–3)	95%
Sakka et al. [40]	2010	Prospective case–control study	106	68	Higher systolic and diastolic BP and TRG.		

## Data Availability

Not applicable.
